# Brain structural differences between fibromyalgia patients and healthy control subjects: a source-based morphometric study

**DOI:** 10.1038/s41598-025-01070-7

**Published:** 2025-05-20

**Authors:** Elijah Agoalikum, Hongzhou Wu, Benjamin Klugah-Brown, Michael Maes

**Affiliations:** 1 Sichuan Provincial Center for Mental Health, Sichuan Provincial People’s Hospital, School of Medicine, University of Electronic Science and Technology of China, Chengdu, 610072 China; 2 Key Laboratory of Psychosomatic Medicine, Chinese Academy of Medical Sciences, Chengdu, 610072 China; 3 The Clinical Hospital of Chengdu Brain Science Institute, MOE Key Laboratory for Neuroinformation, School of Life Science and Technology, University of Electronic Science and Technology of China, No.2006, Xiyuan Avenue, West Hi-Tech Zone, Chengdu, 611731 Sichuan China

**Keywords:** FM, Pain catastrophizing, SBM, Chronic pain, MRI, Components, Neurophysiology, Neuroscience, Medical research

## Abstract

Fibromyalgia (FM) is a chronic pain condition that predominantly affects women. Evidence implies that FM is associated with dysfunction of the central nervous system (CNS). In this study, we investigated the structural differences between FM patients and healthy control (HC) subjects using a multivariate approach. Source-based morphometry (SBM) was applied to structural magnetic resonance imaging (sMRI) data consisting of 20 FM patients (46.4 ± 12.5), and age and gender-matched 20 HC subjects (42.1 ± 12.5). SBM revealed greater grey matter volume (GMV) in the bilateral thalamus in FM patients. Conversely, lower GMV was found in the bilateral putamen, bilateral pallidum, right cerebellum, right calcarine, right amygdala, and bilateral insula in FM patients. Further analysis indicated that grey matter deficits in the pallidum were significantly associated with pain catastrophizing, pain magnification, rumination, and feelings of helplessness, suggesting a link between structural brain changes and clinical pain metrics. These findings provide new insights into the neurobiological underpinnings of FM, highlighting the role of specific brain regions in pain processing and emotional regulation. The results underscore the potential for targeted therapeutic interventions that address both the neurobiological and psychological aspects of FM, paving the way for more effective management strategies for this complex condition.

## Introduction

Fibromyalgia is a chronic pain condition with a complex etiology that predominantly affects women and is marked by extensive musculoskeletal pain along with sleep, memory, fatigue, and mood issues, affecting between 2 and 3% of people worldwide^1,2^, yet the underlying mechanisms contributing to its pathology remain inadequately understood^3^. This lack of clarity continues to pose significant challenges for effective diagnosis and treatment, leading to a pressing need for advanced neuroimaging techniques that can elucidate the neural correlates of FM.

Conventional neuroimaging approaches have highlighted functional abnormalities in the processing of pain in FM patients, showcasing hyperactivity in response to nociceptive stimuli^4,5^. However, there is an essential gap in our understanding of the structural brain changes associated with FM. While voxel-based morphometry (VBM)^6^ has been employed previously to study deficits of grey matter volume in several disorders either on a voxel-by-voxel or seed-to-voxel scale^7,8^, the advanced multivariate technique of Source-Based Morphometry (SBM) presents a more refined methodology for detecting grey matter volumetric alterations^9^. SBM integrates data from multiple voxels to identify covarying “networks” and conducts statistical analysis on the covariation of these networks across subjects, rather than testing each voxel individually^9^. SBM uses independent component analysis (ICA)^10^ to decompose imaging data into maximally independent components, thereby providing a detailed landscape of structural abnormalities across several brain regions and identifying patterns of common variation between two groups^9,11^. This capability is crucial for identifying specific areas impacted by FM, beyond what traditional methods can reveal.

Moreover, cognitive factors such as pain catastrophizing—characterized by an exaggerated emotional response to perceived pain—have been shown to further complicate the clinical presentation of fibromyalgia^12^. Research indicates that pain catastrophizing not only intensifies pain perception but is also linked to structural brain changes, particularly within regions implicated in emotional regulation and pain processing^13,14^. Herein, we aim to investigate the relationship between grey matter volume (GMV) alterations associated with FM and the cognitive-emotional dimension of pain catastrophizing.

This study specifically targets the structural differences in the brains of FM patients compared to healthy controls, using SBM as a robust technique for identification of these differences. By examining how variations in GMV relate to pain catastrophizing and its subscales—such as rumination, magnification, and helplessness—we intend to elucidate the neurobiological underpinnings of FM. Understanding these relationships may provide new avenues for targeted therapeutic interventions, balancing both pharmacological and psychological treatment strategies tailored to the individual needs of FM patients. This represents not only a significant advancement in our understanding of the disorder but also an innovative step towards enhanced management strategies that could substantially improve patient quality of life. We hypothesized that SBM would reveal robust and significant alterations in GMV in FM patients.

## Materials and methods

### Participants

Data from 20 FM patients and 20 healthy controls (HC) subjects were obtained from a public data set (Available at: https://openneuro.org/datasets/ds001928/versions/1.1.0)^15^. FM patients were recruited from the Hospital General of the Secretaria de Salud and an FM help group, both in the city of Queretaro, Mexico. The inclusion criteria for FM individuals were: (1) age 18 years or older, (2) female, (3) meeting the American College of Rheumatology (ACR) 1990 classification criteria and the ACR 2010 diagnostic criteria for FM by a trained rheumatologist, (4) right-handed, and (5) spontaneous and continuous pain in daily life (Visual Rating Scale [VRS] > 5, average of a month). The exclusion criteria were: (1) inability to move or walk, (2) uncontrolled endocrine problems, (3) neurological diseases (e.g., stroke, epilepsy, traumatic brain injury), (4) auditory problems, (5) pregnancy and/or breastfeeding, and (6) MRI contraindications. HC subjects were enrolled and age-matched to the recruited FM participants. The inclusion criteria were: (1) healthy adult females, (2) right-handed. The exclusion criteria were: (1) acute or chronic pain (e.g., osteoarthritis), (2) pregnancy and/or lactating women, and (3) MRI contraindications. Written informed consent was obtained from each patient before the study, which was conducted following the Declaration of Helsinki. Patients received no compensation for taking part in the study. Ethical permission was obtained from the Bioethics Committee of the Institute of Neurobiology, UNAM Juriquilla, Queretaro, Mexico . FM patients were asked not to use painkillers on the day of testing. HC subjects were screened to ensure none of them experienced any type of pain on the day of testing.

To evaluate pain before MRI scanning, pain intensity was measured only in FM patients, using a verbal rating scale (0 = no pain, 10 = worst pain possible). Both FM and HC subjects answered the following questionnaires: the Spanish version of the Pain Catastrophizing Scale (PCS) to measure thoughts and feelings when experiencing pain^16^, State-Trait Anxiety Inventory (STAI) to measure both immediate (state) and broad (trait) emotional, cognitive, and behavioral elements of anxiety^17^, and Center for Epidemiologic Studies Depression Scale (CESD), a questionnaire for depression and depressive disorder^18^. Helplessness, pain magnification, rumination, pain self-perception, and FM years were also recorded for symptom assessment.

### MRI acquisition

The image acquisition was performed with a 3-T GE Discovery MR750 scanner (HD, GE Healthcare, Waukesha, WI, USA) and a commercial 32-channel head coil array. High-resolution T1-weighted anatomical images were obtained using the FSPGR BRAVO pulse sequence with the following parameters: plane orientation = sagittal; TR = 7.7 ms; TE = 3.2 ms; flip angle = 12^0^; matrix = 256 × 256; FOV = 256 mm^2^; slice thickness = 1.1 mm; number of slices = 168; gap = 0 mm; slice order = interleaved.

### Image preprocessing

High-spatial-resolution T1-weighted MR imaging data were processed using the Computational Anatomy Toolbox (CAT12) in statistical parametric mapping software (SPM12). All T1 images were first checked for artifacts and reoriented to adjust image origins at the anterior commissure. Segmentation was then done to separate the T1 images into grey matter (GM), white matter (WM), and cerebrospinal fluid (CSF) and resampled to a volume image resolution of 3 × 3 × 3 mm^3^. After the data quality and sample homogeneity check, the segmented GM images were smoothed using an 8-mm full width at half maximum Gaussian kernel. Total intracranial volume (TIV) was estimated for each subject. The smoothed GM images were used for subsequent analyses.

### Source based-morphometry (SBM) analysis

First, we used the Minimum Description Length (MDL) criterion to estimate the number of components (K)^19^, which resulted in K = 4 components.

All participants’ pre-processed grey matter images were processed using SBM as implemented in the GIFT toolbox (https://trendscenter.org/software/gift/). SBM was performed using the Infomax algorithm^20^. This process was repeated 20 times within the ICASSO algorithm to enhance component reliability and consistency^21^. All participants’ data were converted into one 40-row participant-by-grey matter data matrix “measure matrix” (40 participants × 698,228 voxels) in which each row is a vectorized image of grey matter from each participant, with the voxels from the 3D image unwrapped into a single row. This matrix was then decomposed into a participant-by-component “mixing matrix” and a component-by-voxel “source matrix”. The mixing matrix expresses the relationship between the 40 subjects and the 4 components, with its rows indicating the degree to which the components contribute to each subject while the columns indicate how each component contributes to the 40 subjects (40 subjects × 4 components). The source matrix however expresses the relationship between the voxels in the brain and the 4 components, with its rows indicating how each component contributes to different brain voxels while the columns indicate the contribution of one brain voxel to each component (4 components × 698,228 voxels). Finally, a two-sample *t*-test was performed on loading coefficients in the mixing matrix to identify components with significant differences between the FM and HC groups.

### Correlation analysis

To determine the relationship between abnormal grey matter regions and FM symptoms metrics, we extracted regions that showed significant grey matter differences between FM patients and HC subjects (voxels from the significant ROIs were extracted from all subjects) and performed a partial correlation analysis using age as a covariate. FM metrics used include; FM years, rumination, pain magnification, STAI scores, CESD scores, pain intensity, pain catastrophizing scale scores, pain self-perception scores, and helplessness. GMV values were residualized and used for the correlation analysis in order to accurately reflect the linear relationship between the variables.

### Statistical analysis

Statistical analysis was done using the mixing matrix. Since the columns of the mixing matrix indicate how each component contributes to the 40 subjects, we applied a two-sample t-test to every column of the mixing matrix to determine which components show significant differences between FM patients and HC subjects. Age and TIV were regressed out as covariates to control for their potential confounding effects. False discovery rate (FDR) was used for multiple comparison correction, thresholding at *p* < 0.05. The identified spatial component maps, representing areas with significant differences between FM patients and HC subjects, were visualized with a threshold of |Z|> 3.

## Results

### Demographic and clinical characteristics

Our study comprised 20 FM patients and 20 HC subjects matched for age and sex. The demographic and clinical information of the participants are shown in Table 1. The two groups did not differ significantly in age. FM patients had a mean disease duration of 5.2 ± 5.0 years and a mean pain intensity of 7.2 ± 1.6 indicating they were in severe pain. Compared to healthy controls, FM patients demonstrated higher scores in pain catastrophizing, helplessness, pain magnification, rumination, pain self-perception, anxiety, and depressive symptoms.

**Table 1:** Data demographics and clinical characteristics.

	FM (n = 20)	HC (n = 20)	*P*-value
Age (years)	46.4 ± 12.5	42.1 ± 12.5	0.28
Disease duration (years)	5.2 ± 5.0	–	–
PI	7.2 ± 1.6	–	–
PCS	27.6 ± 12.5	12.0 ± 10.9	< 0.001
Helplessness	13.3 ± 5.7	4.9 ± 4.9	< 0.001
Magnification	*5.3* ± 3.8	2.6 ± 2.6	< 0.02
Rumination	9.3 ± 4.6	4.5 ± 4.2	< 0.002
PSP	56.1 ± 28.7	17.6 ± 23.7	< 0.001
STAI	52.8 ± 20.0	26.1 ± 10.7	< 0.001
CESD	30.6 ± 13.7	11.0 ± 8.6	< 0.001

FM, fibromyalgia; HC, healthy controls; PI, pain intensity; PCS, pain catastrophizing scale; PSP, pain self-perception scale; STAI, state-trait anxiety inventory; CESD, Centre for Epidemiologic Studies depression scale.

### Source based-morphometry

**Fig. 1:**
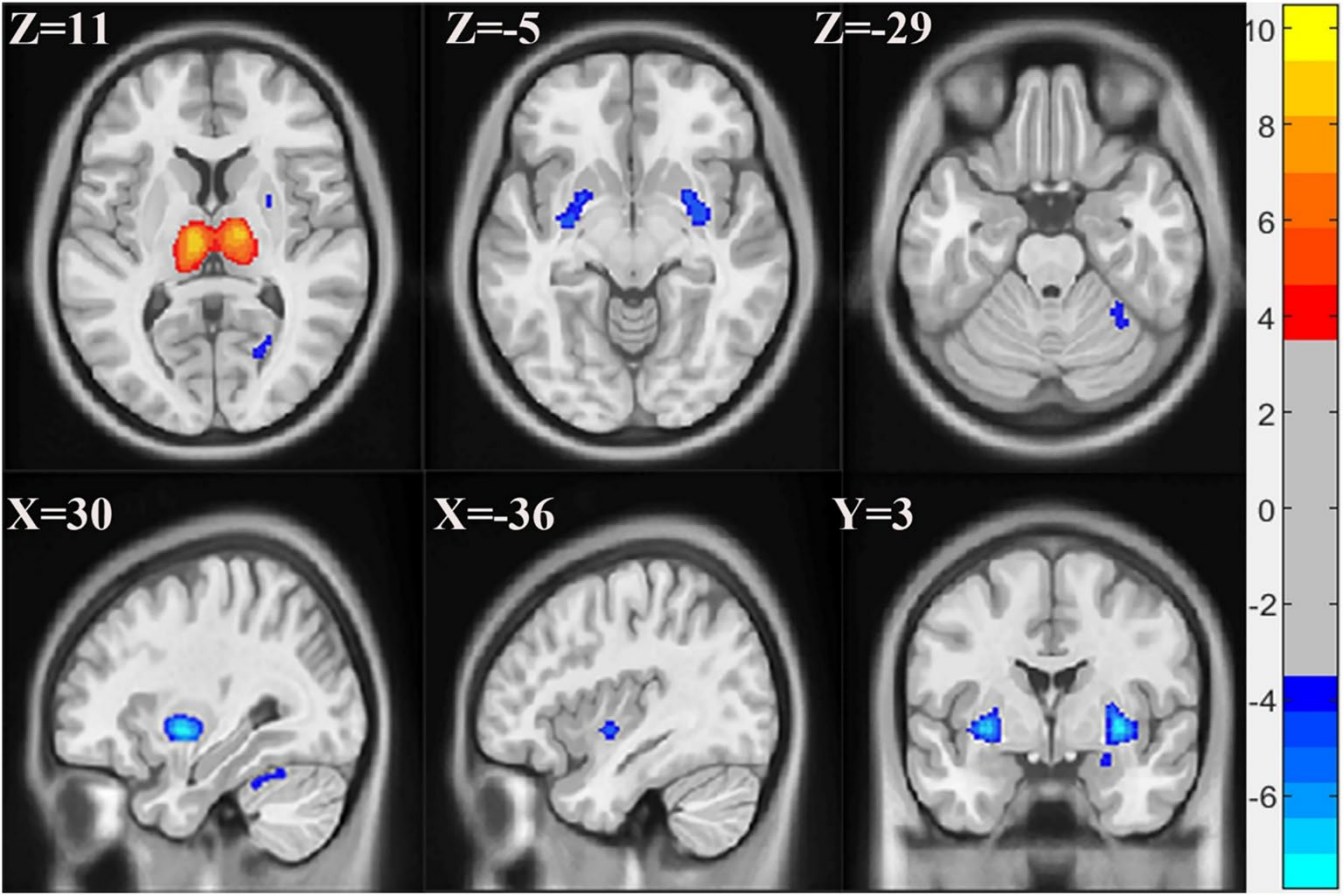
Spatial maps of component 4 showing significant GMV differences between FM patients and HC subjects. Hot (Red) colour represents greater GMV in FM and blue colour represents lower GMV in FM. FDR corrected *P* < 0.001. Spatial maps were shown with a threshold of |Z|> 3. FM, fibromyalgia; HC, healthy control; GMV, grey matter volume; FDR, false discovery rate.

Based on the MDL criterion, the grey matter images of all participants were decomposed into 4 components (Supplementary Fig. 1). All 4 components were stable with no artifacts. Among the 4 components, only component 4 showed significant differences in loading coefficients between FM patients and HC subjects. The patient group showed higher loading coefficients in this component than the HC group (component 4: t = 6.9932, FDR corrected *P* = 0.0002). Components 1, 2, and 3 did not show significant loading coefficients between the 2 groups (component 1: t = − 1.22, *P* = 0.22627; component 2: t = 0.71446, *P* = 0.47931; component 3: t = − 0.53863, *P* = 0.59328). However, the interpretation of loading coefficients depends on the sign of the corresponding spatial map. For instance, if the FM group has higher loading coefficients than the HC group for a component with a predominantly positive spatial map, the FM group exhibits greater tissue volume in this pattern. Conversely, a predominantly negative spatial map indicates rather lower tissue volume in the FM group. The significant component (component 4) representing distinct grey matter regions between the two groups was threshold at |Z|> 3 (Fig. 1). Specifically, the bilateral thalamus showed greater GMV in the FM group while lower GMV in the right cerebellum lobule 6, bilateral putamen, bilateral pallidum, bilateral insula, right amygdala, right hippocampus, and the right calcarine were observed in the FM group. Detailed information about these brain regions is presented in Supplementary Table 1. The insignificant components maps were also threshold at |Z|> 3. Component 1 mainly showed greater GMV in the cerebellum of FM patients compared to HC subjects (Supplementary Fig. 2 and Supplementary Table 2). Component 2 exhibited greater GMV in the bilateral thalamus in FM compared to HC subjects (Supplementary Fig. 3 and Supplementary Table 3). In component 3, the bilateral temporal middle pole, the right hippocampus, left putamen, right fusiform, and left inferior temporal gyrus showed greater GMV in FM patients whiles the right cerebellum lobules 6 and 8 showed lower GMV in FM patients (Supplementary Fig. 4 and Supplementary Table 4).

### Correlation analysis

**Fig. 2:**
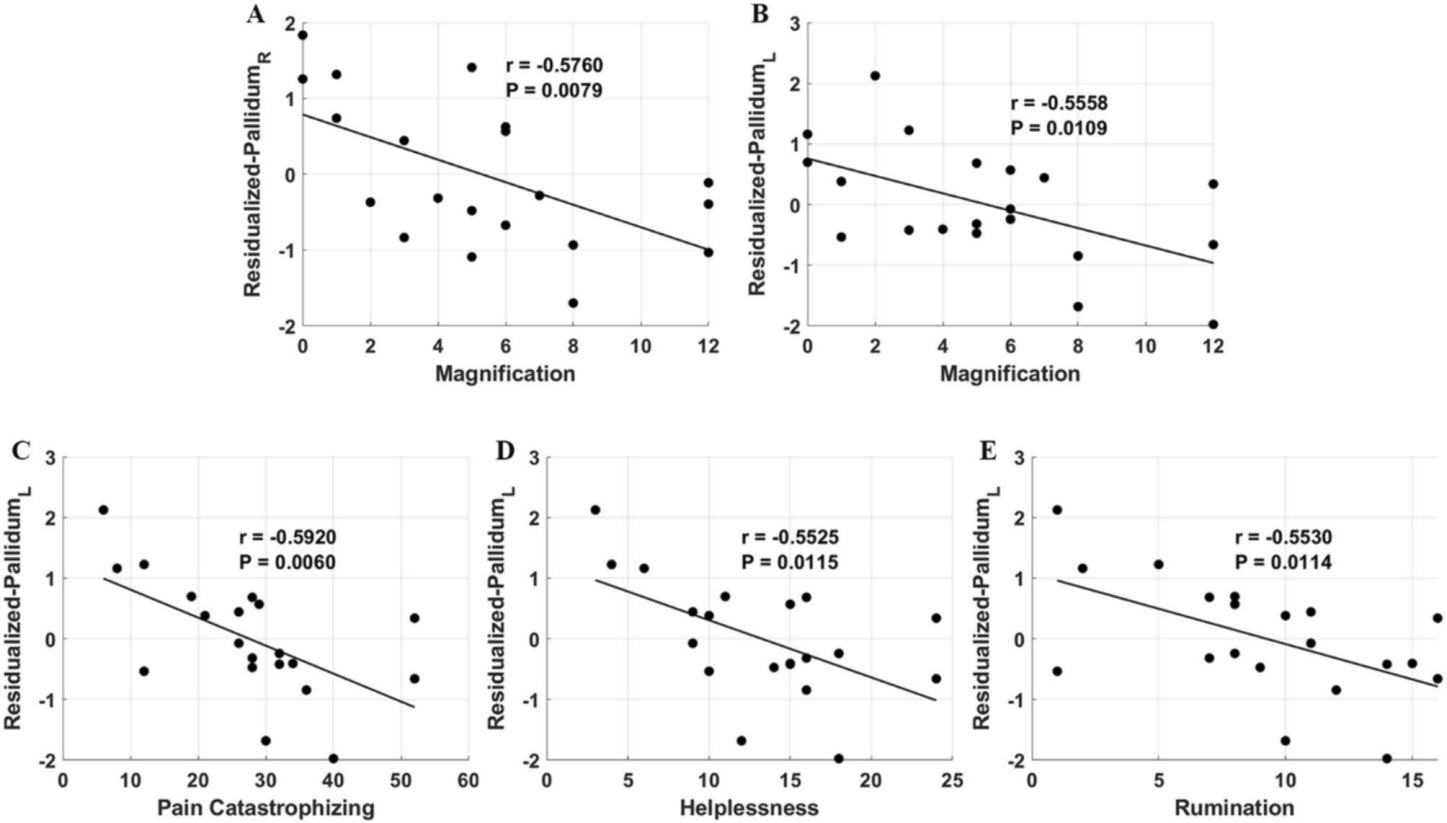
Scatter plots of partial correlation between GMV and FM metrics with age as a covariate. (**A**) Negative correlation between the residualized right pallidum volume and pain magnification. (**B**) Negative correlation between the residualized left pallidum volume and pain magnification. (**C**) Negative correlation between the residualized left pallidum volume and pain catastrophizing. (**D**) Negative correlation between the residualized left pallidum volume and helplessness due to pain. (**E**) Negative correlation between the residualized left pallidum volume and rumination. R, right; L, left; GMV, grey matter volume; FM, fibromyalgia.

To determine the relationship between grey matter anomalies and FM symptom metrics, we used regions that exhibited significant differences between FM and HC subjects in component 4 for correlation analysis. The correlation analysis revealed that grey matter deficits in the right pallidum showed a significant negative relationship with pain magnification (Fig. 2A), while deficits in the left pallidum exhibited significant negative relationships with pain magnification (Fig. 2B), pain catastrophizing (Fig. 2C), helplessness due to pain (Fig. 2D), and rumination (Fig. 2E). This indicates that, a reduction in GMV of the bilateral pallidum results in increases of these FM symptoms.

## Discussion

Our study aimed to enhance understanding of the neurobiological underpinnings of fibromyalgia using magnetic resonance imaging. Our findings revealed significant structural brain differences between FM and HC subjects, including greater GMV in the thalamus and lower GMV in several key brain regions, including the cerebellum, calcarine, pallidum, putamen, insula, amygdala, and hippocampus. Exploratory analysis showed negative correlations between GMV in the pallidum and pain magnification, pain catastrophizing, rumination, and helplessness.

The thalamus plays a critical role in processing nociceptive information, relaying sensory data to cortical areas for further interpretation, and contributing to both the sensory-discriminative and affective-motivational components of pain^22^. Consistent with a previous study^8^, our finding of greater GMV in the thalamus suggests that the thalamic nuclei may be involved in the heightened pain consciousness observed in FM. This suggests a potential mechanism through which FM patients experience amplified pain perception. The cerebellum, traditionally associated with motor control, has also been implicated in higher cognitive functions, including attention, memory, emotion regulation, visual-spatial processing, and learning^23–27^. Furthermore, the cerebellum is involved in nociception^28^. Our finding of lower GMV in the cerebellum aligns with Xin et al.^29^ and may reflect impairments in cognitive control, emotional processing, and pain perception in FM. The cerebellum’s role in both pain modulation and cognitive functions suggests that FM patients may experience difficulties in managing the cognitive and emotional aspects of chronic pain. Compared to HC, we found lower GMV in the bilateral putamen in FM patients. Consistent with our findings, Mosch and colleagues reported decreased putamen volume in FM^8^. The putamen is part of the striatum which is involved in motor function, sensory and pain processing, and goal-directed behaviours that are linked with movement^30^. It is also reported to be involved in cortical input into the basal ganglia and to be activated during pain processing^31^. Therefore, the reduced GMV in this region in FM may indicate impairments in the processing of pain-related motor responses, further contributing to the dysfunction in motor control and sensory processing in FM.

The pallidum, a structure within the basal ganglia, exhibited lower GMV in FM patients in our study. The basal ganglia are involved in modulating emotions and pain perception^32^ and play key roles in the affective dimension of pain, modulation of nociceptive information, sensory gating of nociceptive information to higher motor areas, sensory-discriminative dimension of pain, and cognitive dimension of pain^33^. Pain catastrophizing, characterized by an exaggerated negative mental set during actual or anticipated pain experiences, has been shown to exacerbate pain perception and increase emotional distress^12^. Similarly, the tendency to focus on distressing thoughts has been linked to increased pain sensitivity and emotional distress in chronic pain populations^34^. The correlations between GMV of the pallidum and pain catastrophizing and its subitems suggest that the reduced GMV in the pallidum may be linked to greater pain catastrophizing, magnification, rumination, and feelings of helplessness, with more severe reductions in pallidal GMV corresponding to higher levels of these psychological factors. This aligns with findings from studies showing that pain catastrophizing can magnify pain perception and contribute to the maintenance of chronic pain states^14,35,36^. This highlights the pallidum’s potential role in emotion regulation and the cognitive-affective processing of pain in FM.

Additionally, we found lower GMV in the insula, which is consistent with previous studies^37^. The insula is integral to the integration of diverse sensory modalities and emotional processing^38^. Insula alterations have been reported in several chronic pain conditions and interventions targeting the insula have shown promise in relieving symptoms of chronic pain, making it a key brain area in pain modulation and perception, attention, emotional awareness, and salience detection^39^. While the insula is known to play a critical role in the processing and representation of pain, it is important to acknowledge that its functions extend beyond pain discrimination^38,40^. The insula operates on an anterior–posterior gradient, with different regions associated with distinct aspects of sensory processing and emotional regulation. The anterior insula, for instance, has been implicated in the emotional dimensions of bodily experiences, whereas the posterior insula is more directly involved in the sensory evaluation of pain^40,41^. The reduced GMV of this region and its involvement in both pain and emotion regulation likely contribute to the heightened pain perception and the development of chronic pain and further underscores its importance in FM. Consistent with Lutz et al.^3^, the amygdala showed lower GMV in FM in the current study. The amygdala is involved in the processing of emotions and memory related to fear and has been reported to be activated by pain-related fear^42^. Energy, physical health, pain, fatigue, and symptom distress were improved in FM and chronic pain patients after amygdala retraining^43^, indicating that this region plays an important role in the pathology of FM. This reduction may reflect impairments in emotional regulation, especially concerning fear and anxiety, which are often elevated in FM. Lower GMV of the hippocampus in the current study was also reported in previous studies^3,44^. This brain region is involved in memory, learning, and stress response regulation. It has been reported that stress-induced, *N*-methyl-d-aspartate (NMDA) receptor-mediated dysfunction within the hippocampus has been implicated in the etiopathogenesis and clinical phenomena of FM^45^. This further proves the involvement of the hippocampus in the etiology of fibromyalgia, potentially through its role in memory processing and stress response regulation. The calcarine, a region in the occipital lobe involved in visual processing, also exhibited reduced GMV in FM. Altered activity in the calcarine has been linked to chronic pain conditions^46^, and treatments that target this region have been shown to reduce pain-related activity^47^, indicating the implication of this region in chronic pain. While the precise role of the calcarine in FM remains unclear, its involvement in pain processing may be related to visual-spatial processing mechanisms or even secondary effects of FM-related neural dysfunction.

Several underlying mechanisms may be the cause of these grey matter volume alterations observed in FM patients. Research suggests that neuroinflammation may contribute to the development of FM. Increased levels of pro-inflammatory cytokines have been observed in FM, which could influence brain structure and function^48,49^. Neurotransmitters are involved in developing and maintaining chronic pain through the activation or deactivation of nociceptive neurons in the central nervous system^50^. Therefore, changes in neurotransmitter systems involved in pain modulation may lead to changes in grey matter in pain-related brain regions. Also, chronic pain disorders such as fibromyalgia are connected to neuroplastic alterations in the brain^51^. Prolonged nociceptive input can lead to changes in brain structure and function, including increased excitability and decreased inhibitory control in pain-processing regions^51^. The reductions in GMV observed may reflect a maladaptive response to chronic pain, where the brain’s ability to process and modulate pain signals is compromised. Neurobiological changes associated with FM can be aggravated by genetic vulnerability and environmental factors like prolonged stress or trauma. Bevers and colleagues reported that individuals with a family history of chronic pain disorders may stand a higher chance of developing FM, indicating a genetic component in the condition^52^. Cognitive and emotional factors can also influence brain structure and function. The observed negative relationships between psychometric measures like pain catastrophizing, pain magnification, rumination, and helplessness due to pain and GMV deficits suggest that cognitive and emotional factors play an important role in the pathophysiology of FM. These factors can magnify pain perception and contribute to the maintenance of chronic pain states^35^.

Even though we reported robust structural alterations in FM patients, several limitations should be considered when interpreting the results. One of the main limitations of the study is the relatively small sample size. While our findings are promising, a larger sample would increase the statistical power and help validate these results. Many FM patients are prescribed various medications to manage their symptoms. These medications may potentially affect brain structure, but it is difficult to control their effects. Future research should account for the influence of pharmacological treatments to determine whether the observed structural brain changes are due to FM or potentially confounded by medication use.

In conclusion, the current study investigated structural alterations in FM using a multivariate method. This method revealed robust GMV differences between FM and HC subjects, with the bilateral thalamus exhibiting greater GMV in FM while lower GMV in FM was found in the cerebellum, putamen, pallidum, hippocampus, amygdala, calcarine, and insula. Grey matter deficits in the pallidum exhibited a negative relationship with pain catastrophizing and its subscales. However, understanding these brain alterations has significant clinical implications for the management and treatment of FM. The identified brain areas associated with FM symptoms suggest that treatment approaches could be tailored to target these areas. For example, interventions such as cognitive-behavioural therapy (CBT) could be designed to address maladaptive thought patterns (e.g., pain catastrophizing) that correlate with grey matter deficits in the pallidum. CBT has been shown to reduce pain and improve coping strategies in chronic pain populations, including FM^53^. Also, our findings may inform pharmacological treatments. For instance, medications that target neurotransmitter systems involved in pain processing, such as serotonin and norepinephrine reuptake inhibitors (SNRIs), could be particularly effective in patients exhibiting significant grey matter deficits in pain-related brain regions. It has been previously shown that SNRIs can reduce pain and improve brain function in FM patients^54^. Neurofeedback techniques could also be explored as a therapeutic option aiming to train individuals to alter their brain activity, potentially leading to improvements in pain perception and emotional regulation. Research suggests that this technique can enhance self-regulation of brain function and may be beneficial for chronic pain conditions^55^. Our work further provides a comprehensive understanding of the pathogenesis and the evidence of a subcortical-cortical pain processing system in FM patients, where nociceptive information is processed and relayed from subcortical regions through the thalamus to cortical areas.

## Supplementary Information

Supplementary Information.

## Data Availability

The data used for this study are available at https://openneuro.org/datasets/ds001928/versions/1.1.0.
