# Supplementary material for: Brain structural differences between fibromyalgia patients and healthy control subjects: a source-based morphometric study
Source: Sci Rep. 2025 May 20;15:17446. doi: 10.1038/s41598-025-01070-7 (PMC12092787; doi:10.1038/s41598-025-01070-7)
Supplement: Supplementary file 1 — Supplementary Information. [file 41598_2025_1070_MOESM1_ESM.pdf]

Supplementary Material

Brain Structural Differences Between Fibromyalgia Patients and Healthy Control Subjects: A Source-Based Morphometric Study.

Elijah Agoalikum[12*], Hongzhou Wu[12], Benjamin Klugah-Brown[3], Michael Maes[12*]

1 Sichuan Provincial Center for Mental Health, Sichuan Provincial People's Hospital,

School of Medicine, University of Electronic Science and Technology of China,

Chengdu 610072, China

2 Key Laboratory of Psychosomatic Medicine, Chinese Academy of Medical Sciences,

Chengdu 610072, China.

3 The Clinical Hospital of Chengdu Brain Science Institute, MOE Key Laboratory for Neuroinformation, School of Life Science and Technology, University of Electronic Science and Technology of China, No.2006, Xiyuan Avenue, West Hi-Tech Zone, Chengdu, Sichuan 611731, China.

*Correspondence:

Elijah Agoalikum
agoalikumelijah@gmail.com

Michael Maes
dr.michaelmaes@hotmail.com

Address: Sichuan Provincial Center for Mental Health, Sichuan Provincial People's Hospital, School of Medicine, University of Electronic Science and Technology of China, Chengdu 610072, China.

Telephone: +8619381980441

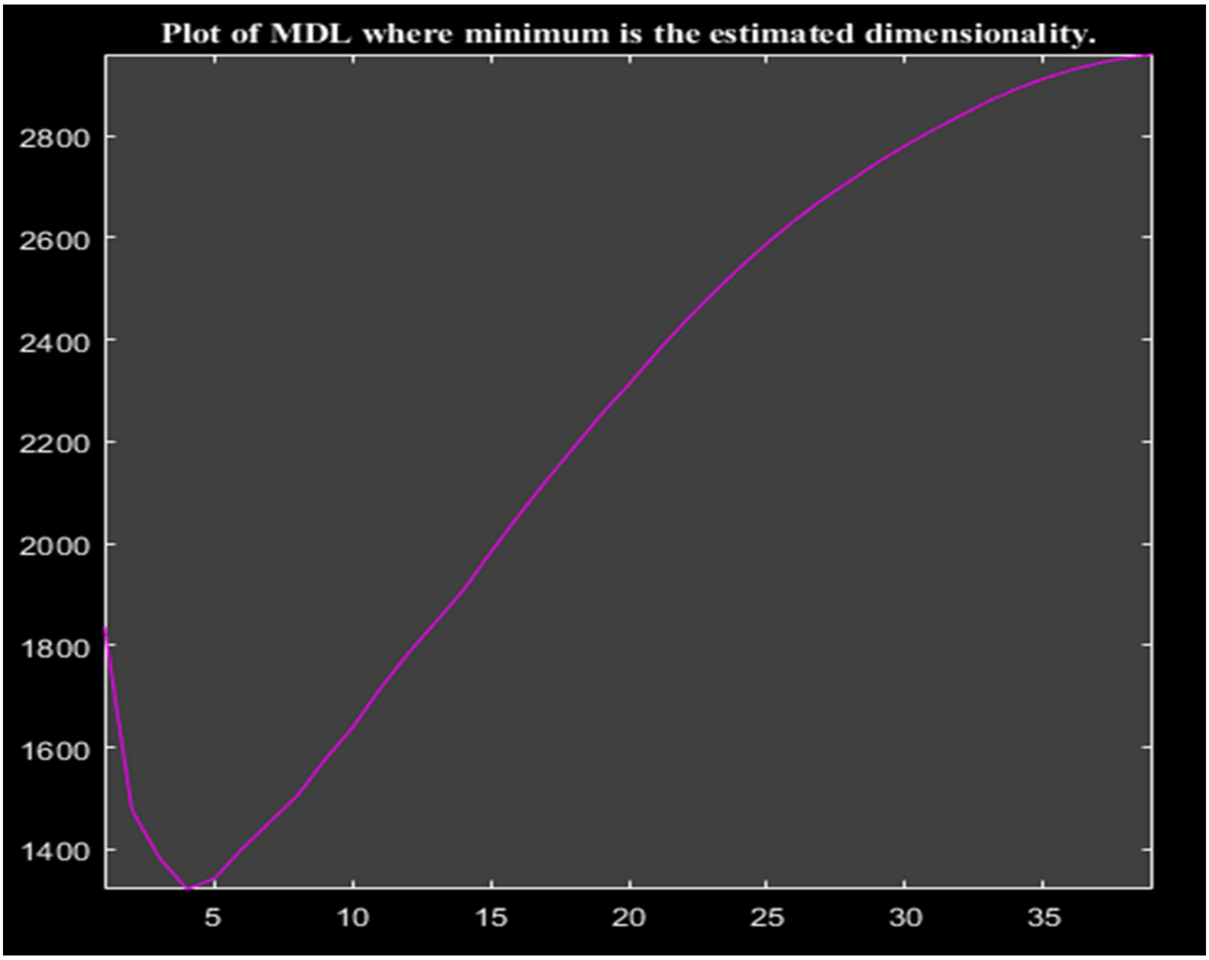

Supplementary Figure 1: The estimated independent components were identified to be 4 using the Minimum Description Length (MDL) criterion.

Supplementary Table 1: Brain regions showing GMV differences between FM patients compared and HC subjects in the significant component 4.

Brain Region	MNI Coordinates	GMV alteration
Thalamus_L	-9 -17 11	greater
Thalamus_R	10 -13 11	greater
Cerebelum_6_R	33 -42 -29	lower
Putamen_R	30 0 -3	lower
Putamen_L	-32 -6 -5	lower
Pallidum_R	24 3 1	lower
Pallidum_L	-25 -1 -2	lower
Insula_R	40 -5 -5	lower
Insula_L	-36 -6 -5	lower
Amygdala_R	24 3 -12	lower
Hippocampus_R	24 -2 -19	lower
Calcarine_R	21 -69 9	lower

L = Left; R = Right; MNI = Montreal Neurological Institute; GMV = Grey Matter Volume.

Supplementary Table 2: Brain regions showing GMV differences between FM patients compared and HC subjects in the insignificant component 1.

Brain Region	MNI Coordinates	GMV alteration
Cerebelum_8_L	-20 -60 -54	greater
Cerebelum_8_R	19 -58 -51	greater
Cerebelum_6_R	21 -65 -21	greater
Cerebelum_6_L	-18 -65 -20	greater

L = Left; R = Right; MNI = Montreal Neurological Institute; GMV = Grey Matter Volume.

Supplementary Table 3: Brain regions showing GMV differences between FM patients compared and HC subjects in the insignificant component 2.

Brain Region	MNI Coordinates	GMV alteration
Thalamus_R	8 -17 9	greater
Thalamus_L	-7 -18 9	greater

L = Left; R = Right; MNI = Montreal Neurological Institute; GMV = Grey Matter Volume.

Supplementary Table 4: Brain regions showing GMV differences between FM patients compared and HC subjects in the insignificant component 3.

Brain Region	MNI Coordinates	GMV alteration
Temporal_Pole_Mid_R	40 17 -38	greater
Temporal_Pole_Mid_L	-42 17 -33	greater
Hippocampus_R	27 -11 -21	greater
Putamen_L	-29 2 -3	greater
Fusiform_R	29 -6 -40	greater
Temporal_Inf_L	-53 -12 -35	greater
Cerebelum_8_R	39 -47 -47	lower
Cerebelum_6_R	36 -53 -29	lower

L = Left; R = Right; Mid = Middle; Inf = Inferior; MNI = Montreal Neurological Institute; GMV = Grey Matter Volume.

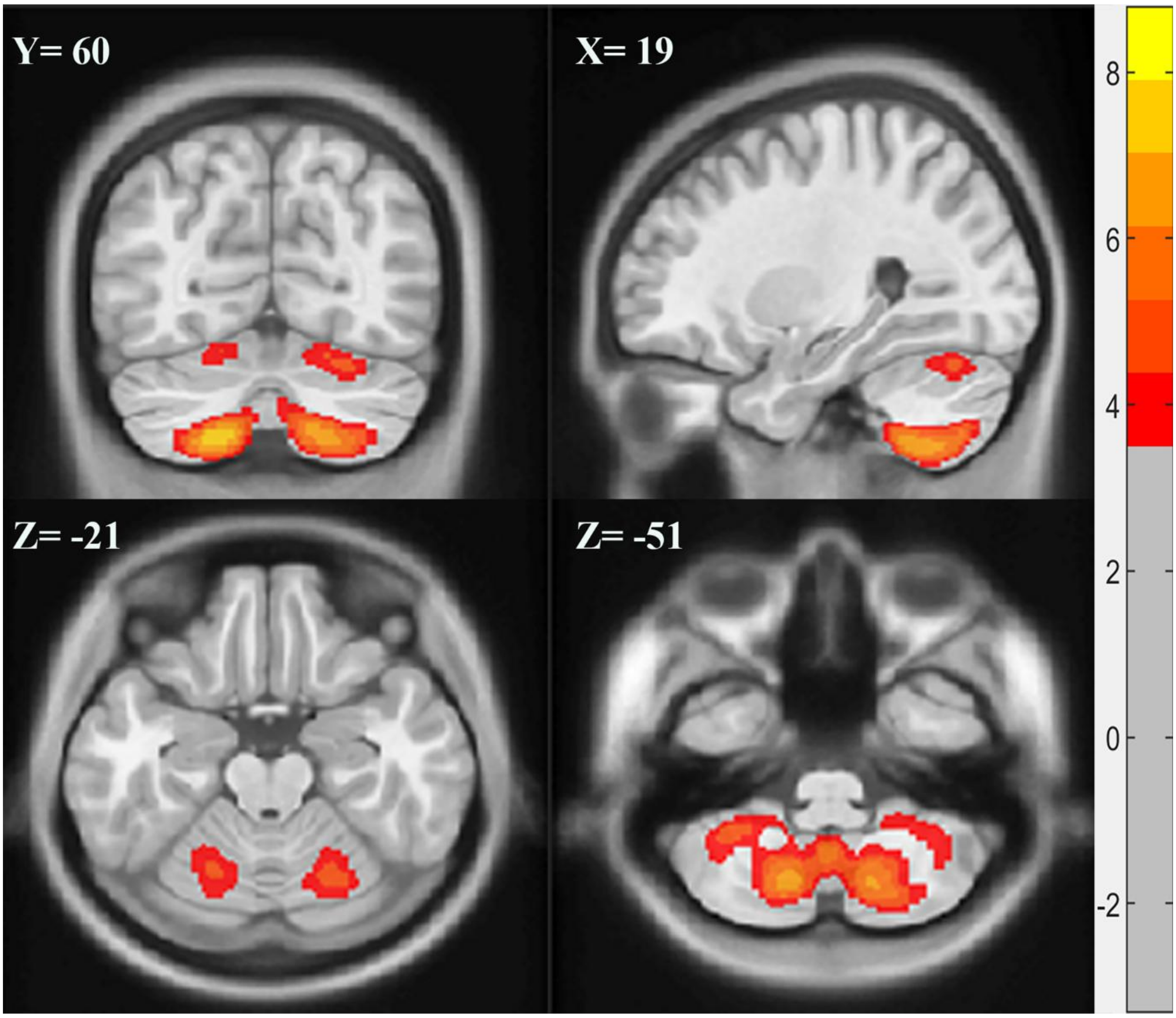

Supplementary Figure 2: Spatial maps of component 1 showing GMV differences between FM patients and HC subjects. FM patients showed greater GMV in this insignificant component than HC subjects. Spatial maps were shown with a threshold of |Z| > 3. GMV = Grey Matter Volume; FM = Fibromyalgia; HC = Healthy Control.

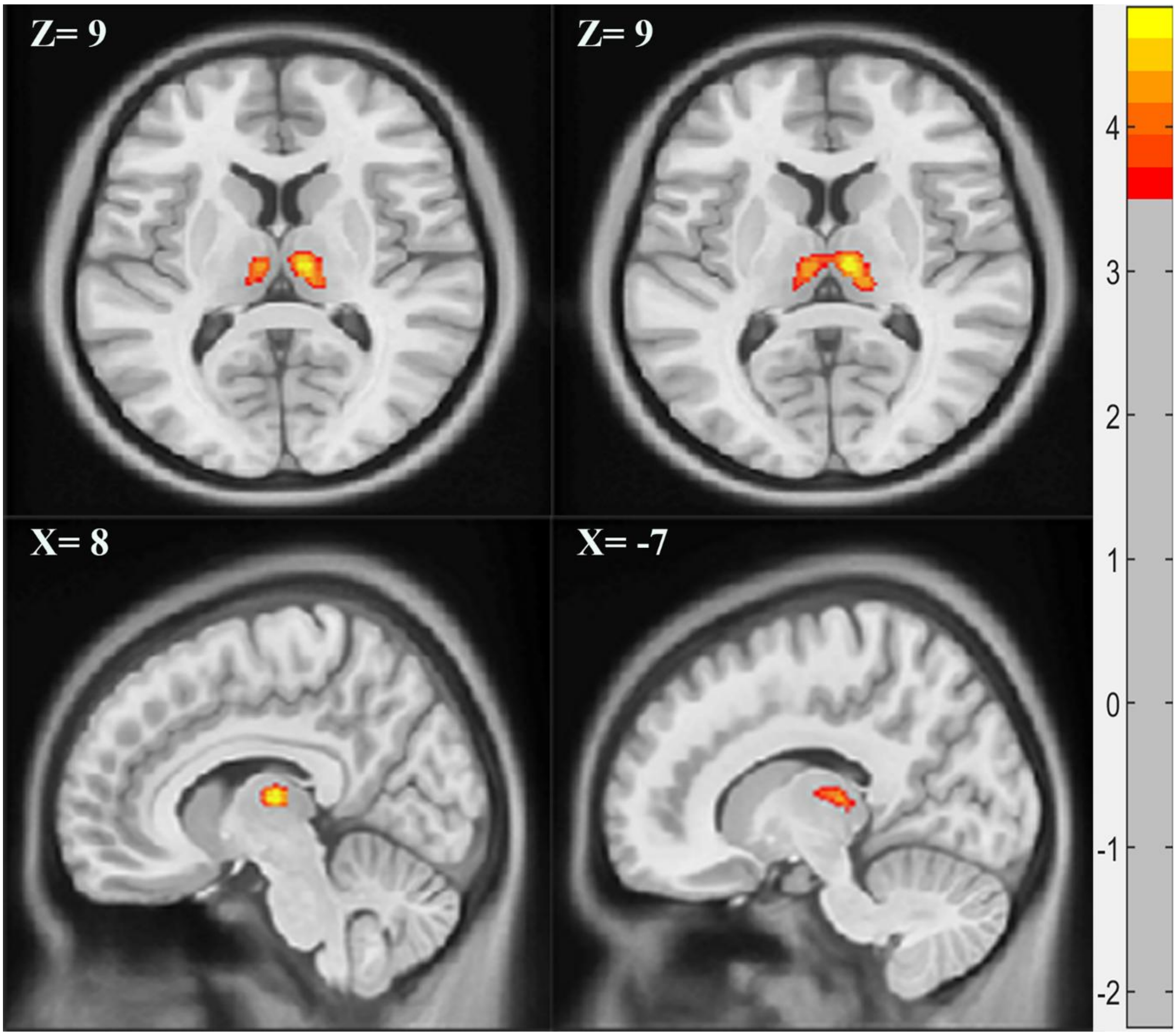

Supplementary Figure 3: Spatial maps of component 2 showing GMV differences between FM patients and HC subjects. FM patients showed greater GMV in this insignificant component than HC subjects. Spatial maps were shown with a threshold of |Z| > 3. GMV = Grey Matter Volume; FM = Fibromyalgia; HC = Healthy Control.

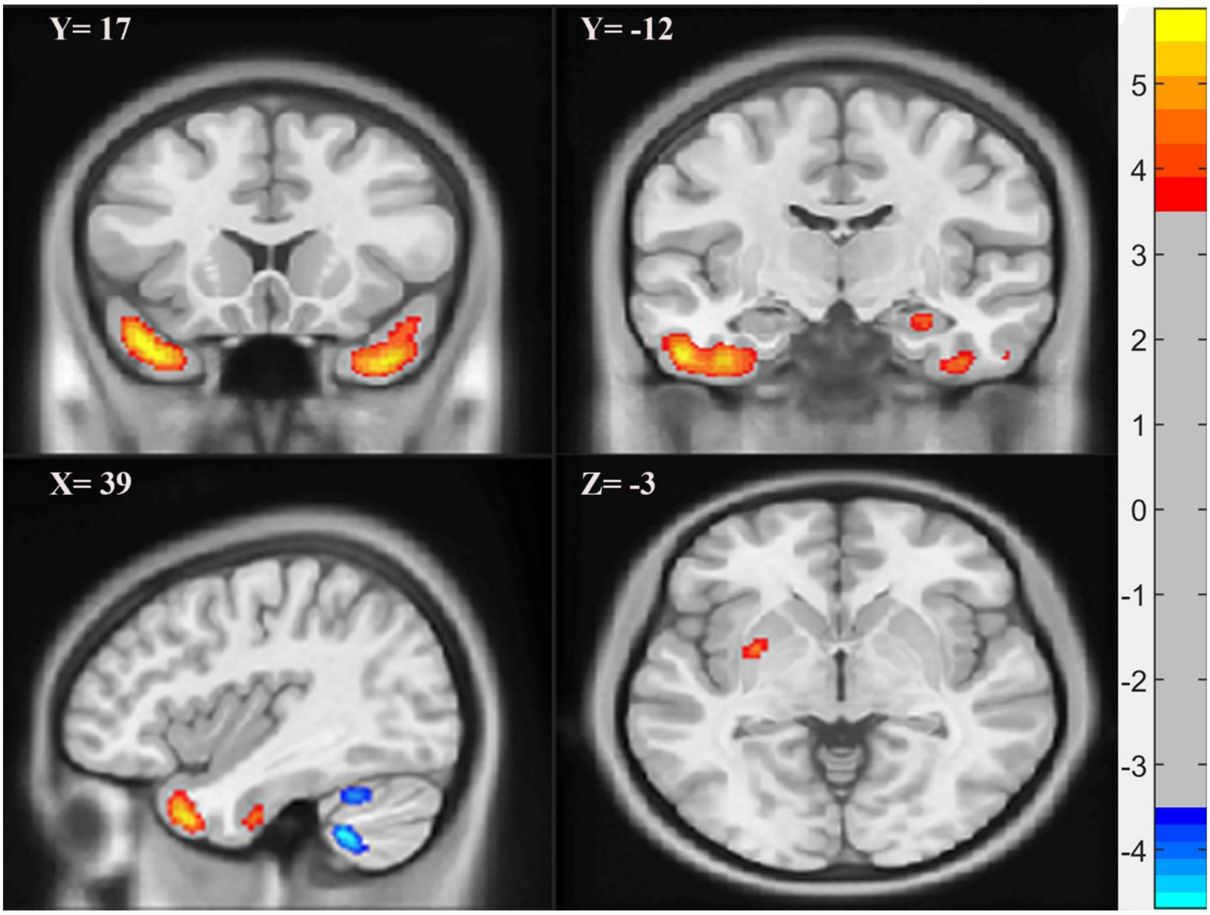

Supplementary Figure 4: Spatial maps of the insignificant component 3 showing GMV differences between FM patients and HC subjects. Hot (Red) colour represents greater GMV in FM and blue colour represents lower GMV in FM. Spatial maps were shown with a threshold of |Z| > 3. GMV = Grey Matter Volume; FM = Fibromyalgia; HC = Healthy Control.
